# Exosome‐Delivered eNAMPT From Exercise Activates SIRT1 to Counteract Age‐Related Hepatic Steatosis and Fibrosis

**DOI:** 10.1111/acel.70541

**Published:** 2026-05-10

**Authors:** Wenxuan Song, Naijun Wu, Xing Li, Dan Li, Jiaqi Li, Siqi Liu, Qiang Ma, Zhiwei Yue, Xuefeng Zhu, Yajuan Qi

**Affiliations:** ^1^ School of Basic Medical Sciences North China University of Science and Technology Tangshan China; ^2^ Hebei Key Laboratory of Basic Medical Sciences for Chronic Diseases Tangshan China; ^3^ Tangshan Key Laboratory of Basic Research in Medicine Development North China University of Science and Technology Tangshan China; ^4^ Department of Endocrinology North China University of Science and Technology Affiliated Hospital Tangshan China; ^5^ School of Pharmacy North China University of Science and Technology Tangshan China

**Keywords:** autophagy, epithelial‐mesenchymal transition, exercise, exosomes, metabolic‐related fatty liver disease, SIRT1

## Abstract

Aging is a major independent risk factor for the development and progression of metabolic dysfunction‐associated steatotic liver disease (MASLD); however, effective therapeutic strategies for this population remain limited. Here, we established a model of aging‐associated MASLD by subjecting aged mice to a long‐term high‐fat diet (HFD), which recapitulated key disease features including progressive hepatic steatosis, inflammation, insulin resistance, and fibrosis. A 6‐week exercise intervention markedly ameliorated these pathologies by restoring insulin sensitivity and suppressing TGF‐β/Smad‐mediated fibrotic signaling. We identified exercise‐derived exosomes (Exercise‐Exos) as primary mediators of these benefits. Western blot analysis revealed that extracellular nicotinamide phosphoribosyltransferase (eNAMPT) was markedly enriched in Exercise‐Exos compared to those from sedentary controls. Delivery of exosomal eNAMPT activated the hepatic SIRT1‐autophagy axis, restored autophagic flux, and inhibited epithelial‐mesenchymal transition (EMT). These effects were demonstrated by an increased LC3‐II/LC3‐I ratio, reduced p62 accumulation, downregulation of mesenchymal markers (α‐SMA, Vimentin), and upregulation of the epithelial marker *E*‐cadherin. Furthermore, Exercise‐Exos treatment significantly reduced collagen deposition. Critically, all protective effects were abolished upon pharmacological inhibition of SIRT1 with EX‐527, establishing the necessity of the eNAMPT‐NAD^+^‐SIRT1 cascade. Collectively, our results elucidate a novel exosome‐mediated pathway through which exercise mitigates age‐related liver disease, positioning exosomal eNAMPT as a promising therapeutic agent and exercise mimetic for MASLD.

## Graphical abstract

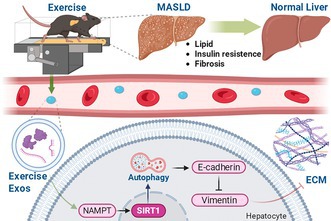

## 1 Introduction

Metabolic dysfunction‐associated steatotic liver disease (MASLD) is a highly prevalent chronic liver disease globally, with its incidence increasing in tandem with population aging and obesity. Aging is a major risk factor for MASLD progression, due to age‐related metabolic decline, insulin resistance, and diminished autophagic flux (Gan et al. 2011; I. H. Kim et al. 2015). Although exercise is well‐established as a beneficial intervention for improving metabolic health (Chomiuk et al. 2024), its capacity to mitigate age‐associated hepatic injury under the sustained challenge of a prolonged high‐fat diet (HFD) remains poorly characterized. Whether exercise can reverse irreversible damage and restore hepatocellular homeostasis in aged livers requires further investigation.

SIRT1, an NAD^+^‐dependent deacetylase, is a pivotal regulator of metabolic homeostasis and longevity. It sustains hepatic health through dual mechanisms: first, by enhancing autophagic flux via the deacetylation of core autophagy proteins such as LC3 to promote autophagosome maturation; and second, by concurrently suppressing fibrotic processes through inhibition of the TGF‐β/Smad pathway, thereby attenuating epithelial‐mesenchymal transition (EMT) (Sah et al. 2025; Huang et al. 2015; Lu et al. 2023; Zerr et al. 2016). However, aging and chronic HFD exposure potently suppress hepatic SIRT1 expression. This downregulation initiates a vicious pathogenic cycle: diminished SIRT1 activity impairs autophagic clearance, exacerbating hepatic lipid accumulation and oxidative stress. Concurrently, the loss of SIRT1‐mediated repression of TGF‐β/Smad signaling promotes sustained EMT activation, which drives progressive fibrosis and the disruption of normal hepatic architecture (Ren et al. 2024; Wang, Ping, et al. 2024; Wang, Ren, et al. 2024; Xu et al. 2020).

Emerging evidence highlights exercise‐derived exosomes (Exercise‐Exos) as novel mediators of inter‐tissue communication, capable of reprogramming systemic metabolism (Whitham et al. 2018). Extracellular vesicles are enriched with bioactive molecules, including extracellular nicotinamide phosphoribosyltransferase (eNAMPT)—a rate‐limiting enzyme in the NAD^+^ salvage pathway (Yoshida et al. 2019). The delivery of eNAMPT via Exercise‐Exos is known to enhance SIRT1 activity in distant target organs, underpinning their documented protective effects in models of young, healthy animals (Chong et al. 2022; Morató et al. 2022; Yoshioka et al. 2025). While Exercise Exos have demonstrated protective effects in young animals, their capacity to rejuvenate senescent hepatocytes and reverse fibrotic progression in the context of the aged liver remains unclear.

In this study, we investigated the therapeutic potential of exercise‐derived exosomes (Exercise‐Exos) against MASLD in aging. Using a long‐term HFD fed aged mouse model and an in vitro model of hepatocyte senescence, we demonstrate that Exercise‐Exos deliver eNAMPT to senescent hepatocytes. This delivery reactivates the SIRT1‐autophagy axis, suppresses EMT, and ultimately attenuates hepatic steatosis and fibrosis. Our findings reveal a novel exosome‐mediated mechanism through which exercise confers its benefits, highlighting a potential therapeutic avenue for counteracting age‐related liver disease.

## 2 Materials and Methods

### 2.1 Animals

A total of 48 male SPF‐grade C57BL/6J mice (10 months) were obtained from Huafukang Biotechnology. (SCXK 2020‐007). All animal experiments were conducted humanely, following the National Research Council's Guide for the Care and Use of Laboratory Animals, and were approved by the North China University of Science and Technology (Approval No. 2023‐SY‐227). This study is reported in accordance with the ARRIVE guidelines. Mice were housed in the university's animal facility under controlled environmental conditions (22°C–28°C) with a 12‐h light/dark cycle and free access to food and water.

**FIGURE 1:**
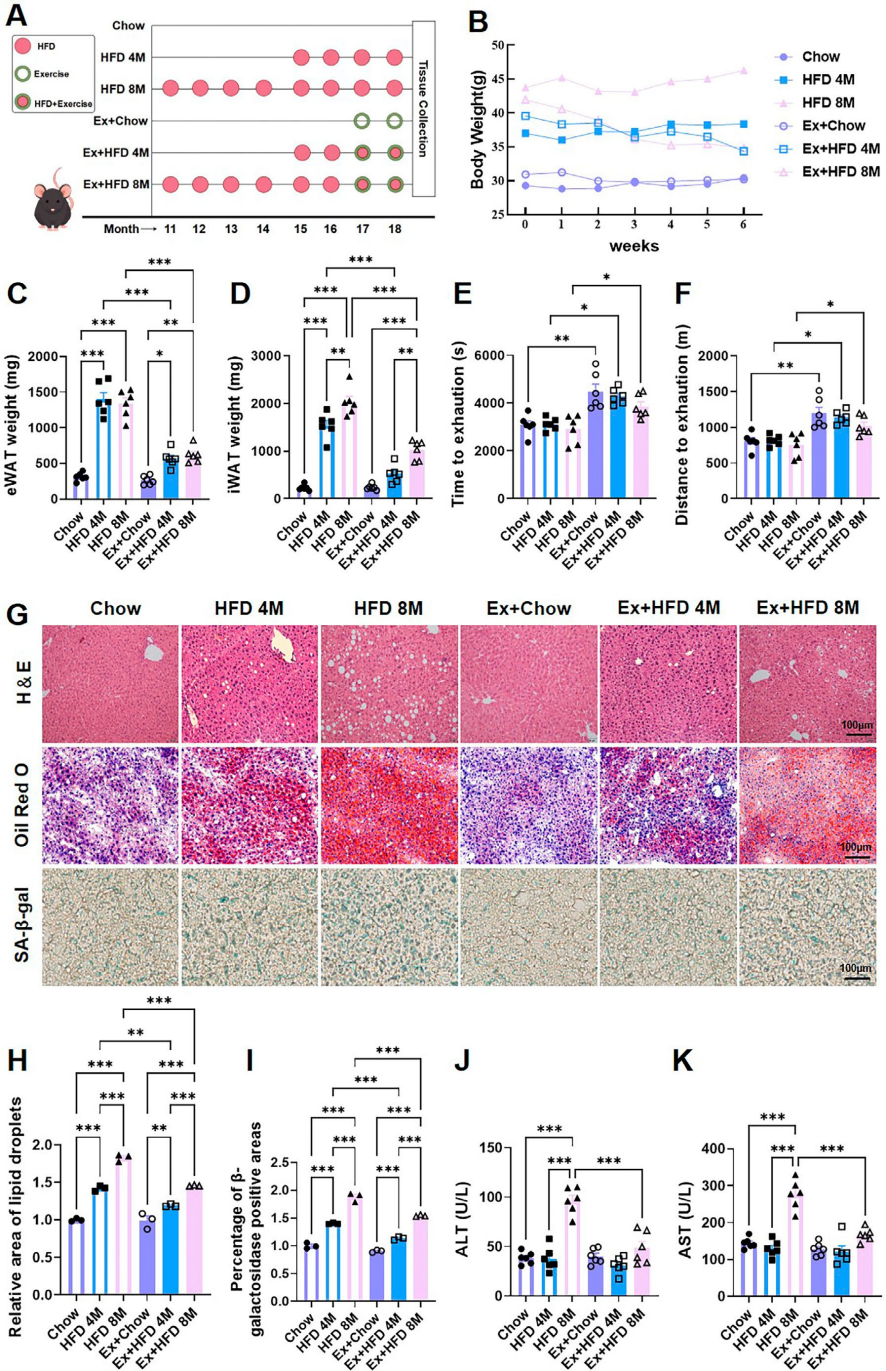
Effects of high‐fat diet and exercise intervention on metabolic and physiological phenotype in aged mice. (A) Schematic overview of the experimental design showing mouse age, duration of high‐fat diet (HFD) feeding, timing of exercise intervention, and tissue collection. (B) Changes in body weight during the 6‐week exercise intervention period (*n* = 6). (C, D) Weights of epididymal (eWAT) and inguinal (iWAT) white adipose tissues (*n* = 6). (E, F) Physical performance capacity measured by time to exhaustion and distance to exhaustion following exercise intervention (*n* = 6). (G‐I) Representative liver histological staining and corresponding quantification: Hematoxylin and eosin (H&E) staining for morphology, Oil Red O staining for lipid accumulation, and senescence‐associated β‐galactosidase (SA‐β‐gal) staining for cellular senescence (*n* = 3, scale bars: 100 μm, 200× objective). (J, K) Serum alanine aminotransferase (ALT) and aspartate aminotransferase (AST) levels as indicators of liver injury (*n* = 6). Data are presented as mean ± SEM. Statistical significance was determined by two‐way ANOVA followed by Tukey's multiple‐comparisons test. *p < 0.05, **p < 0.01, ***p < 0.001.

As depicted in Figure 1A, thirty‐six 11‐month‐old mice were fed a HFD to establish an aging‐associated MASLD model, after which a subset of mice underwent an exercise intervention. Mice were randomly assigned to six groups: (1) Chow group: fed a standard chow diet throughout the entire study period; (2) HFD 4 M group: fed an HFD for 4 months; (3) HFD 8 M group: fed an HFD for 8 months; (4) Ex+Chow group: maintained on a standard chow diet and subjected to 6 weeks of exercise training; (5) Ex+HFD 4 M group: fed an HFD for 4 months with concurrent 6‐week exercise training; and (6) Ex+HFD 8 M group: fed an HFD for 8 months with concurrent 6‐week exercise training. All animals were euthanized at 18 months of age for tissue collection and analysis.

### 2.2 Body Weight and Blood Glucose Monitoring

Body weight was measured weekly at a fixed time (9:00 AM), with animals allowed *ad libitum* access to food and water. Blood glucose levels were assessed during the final week of the study, prior to tissue collection. All glucose measurements were performed at 9:00 AM to control for circadian variation. Both non‐fasting (ad libitum) and fasting blood glucose were measured. For fasting glucose, food was removed for 12 h (overnight) prior to measurement, with free access to water. Blood was collected from the tail vein and glucose concentration was determined using a portable glucometer (Johnson & Johnson, CNHBC58, China).

### 2.3 Serum Sample Collection and Processing

At the experimental endpoint, mice were anesthetized with isoflurane, and whole blood was collected via the retro‐orbital venous plexus. Blood samples were allowed to clot at room temperature for 1 h, and then kept at 4°C for an additional 2 h. Samples were subsequently centrifuged at 4°C at 3000 rpm for 10 min (Eppendorf, 5920R, Germany). The resulting serum supernatant was aliquoted and stored at −80°C for subsequent analysis.

### 2.4 Exercise Training Protocol

The exercise protocol was slightly modified based on previously published methods (C. He et al. 2012; Kuramoto et al. 2023). Acute exercise adaptation: To acclimate the mice to treadmill running, mice underwent a two‐day adaptation phase. On Day 1, they ran at a speed of 8 m/min for 5 min. On Day 2, they ran at 8 m/min for 5 min followed by 10 m/min for another 5 min. On Day 3, acute treadmill running was initiated at a starting speed of 12 m/min for 40 min. After this initial period, the speed was increased by 1 m/min every 10 min for the next 30 min, and subsequently by 1 m/min every 5 min for an additional 20 min.

Chronic exercise training: Following the acute adaptation phase, mice began a 6‐week treadmill training regimen. The session started at a speed of 8 m/min, with the speed increasing by 2 m/min every 2 min until reaching a maximum of 16 m/min, which was then maintained for 50 min. The session concluded with a gradual decrement in speed. Throughout all sessions, the treadmill was maintained in a horizontal position (0° incline).

### 2.5 Cell Culture and Treatment

AML12 cells (Shanghai Zhong Qiao Xin Zhou Biotechnology, China) and mouse primary hepatocytes were used in this study. Primary hepatocytes were isolated and purified according to the protocol described in reference (Charni‐Natan and Goldstein 2020). Cells were cultured in Dulbecco's modified Eagle's medium (DMEM) supplemented with 10% fetal bovine serum (FBS), 100 U/mL penicillin, and 100 μg/mL streptomycin. To establish an in vitro model of MASLD, primary hepatocytes were exposed to 0.25 mM free fatty acids (FFA; oleic acid: sodium palmitate = 2:1) and 50 mM D‐galactose (D‐Gal, Yuan Ye, S11050, China) for 24 h as previously described (Kanuri and Bergheim 2013; Sha et al. 2021). After model induction, cells were treated with exercise‐derived exosomes (100 μg/mL) or resting‐derived exosomes (100 μg/mL) for 24 h (Kang et al. 2022). Meanwhile, a specific SIRT1 inhibitor, EX‐527 (Beyotime, SC0281, China) 10 μM, was also used in combination to evaluate the pathway dependency of the Exos effect (Li et al. 2023; Zhang et al. 2021).

### 2.6 Preparation and Characterization of Exosomes

After acute exercise intervention, serum was collected from C57BL/6J mice. According to previous study (Wang, Ping, et al. 2024; Yanagawa et al. 2024), Exos were isolated from serum by differential centrifugation using an ultra‐high‐speed centrifuge (Beckman Coulter, USA) (Figure 6A). Transmission electron microscopy (TEM, Hitachi, Japan) was used to examine the morphology and structural integrity of isolated Exos. The size distribution of Exos was further analyzed using nanoparticle tracking analysis (NTA, Malvern, UK).

### 2.7 Blood Biochemistry Analysis

The hepatic function was assessed by measuring serum ALT and AST levels using commercially available kits (Beijing Ruizheng Shanda Biological Engineering Technology, China) with an automated biochemical analyzer (Beijing Prang New Technology, China).

### 2.8 H&E and Masson Trichrome Staining

Mouse liver tissue was fixed with 4% paraformaldehyde for 24 h, dehydrated with gradient ethanol, embedded in paraffin and prepared into 4‐μm sections for future use. For H&E staining, the sections were dewaxed with xylene, hydrated with gradient ethanol, stained with hematoxylin for 5 min, rinsed with running water to turn blue, stained with eosin for 1 min, rinsed with running water, dehydrated and transparent, and sealed with neutral gum. For Masson trichrome staining (Solarbio, 20,230,224, China), paraffin sections were first dewaxed and hydrated. Subsequently, they were subjected to the following sequential staining procedures: immersion in Weigert's iron hematoxylin solution for 5 min, Ponceau acid fuchsin solution for 5 min, differentiation in phosphomolybdic acid solution for 5 min, and subsequent staining with aniline blue solution for 5 min. The stained sections were then differentiated using 1% glacial acetic acid, followed by dehydration, clearing, and final mounting for microscopic observation.

### 2.9 Oil Red O and SA‐β‐Galactosidase (SA‐β‐Gal) Staining

Fresh liver tissues were embedded in optimal cutting temperature (OCT) compound and immediately snap‐frozen to preserve tissue morphology. Frozen sections with a thickness of 10 μm were prepared for subsequent staining assays. Oil Red O Staining: the sections were fixed with 4% paraformaldehyde for 10 min and then washed with PBS. Next, the sections were treated with 60% isopropanol for 5 min and stained with Oil Red O working solution for 15 min. After staining, the sections were differentiated in 60% isopropanol until the background was clear, counterstained with hematoxylin for 1 min, and mounted with glycerol gelatin. SA‐β‐gal staining: The sections were fixed with 4% paraformaldehyde for 15 min and washed with PBS. Then, the sections were incubated with SA‐β‐galactosidase (Beyotime, C0602, China) staining solution at 37°C in the dark for 16 h. The reaction was terminated with PBS, and the sections were mounted with neutral resin.

### 2.10 Immunohistochemistry (IHC) and Immunofluorescence (IF)

For IHC, dewaxed and rehydrated sections were subjected to antigen retrieval by microwaving in pH 6.0 sodium citrate buffer at 95°C for 15 min, followed by natural cooling to room temperature (RT). Endogenous peroxidase activity was quenched by incubation with 3% H_2_O_2_ for 15 min at RT. Non‐specific binding sites were blocked with 5% normal goat serum containing 0.1% Triton X‐100 for 1 h at RT. Sections were then incubated overnight at 4°C with the following primary antibodies: anti‐α‐SMA (1:200, #22p5934, Affinity Biosciences, USA) and anti‐SIRT1 (1:150, #WL02995, Wanleibio, China). The following day, sections were incubated with an HRP‐labeled secondary antibody (1:500, ZSGB‐BIO, China) for 1 h at RT. Signal detection was performed using a DAB substrate kit (Sigma‐Aldrich, USA) for 5 min, and nuclei were counterstained with hematoxylin. Finally, sections were dehydrated, cleared, and mounted with neutral gum.

For IF, dewaxed and rehydrated sections underwent antigen retrieval by microwaving in 10 mM pH 9.0 EDTA buffer at 95°C for 20 min. Sections were permeabilized with 0.5% Triton X‐100 for 15 min and blocked with 5% normal donkey serum containing 1% BSA for 1 h at RT. Subsequently, sections were incubated overnight at 4°C with primary antibodies against LC3I/II (1:1000, #WL01506, Wanleibio, China) and Vimentin (1:500, #WL01960, Wanleibio, China). The following day, sections were incubated with Alexa Fluor 488‐conjugated anti‐rabbit secondary antibody (1:200, #S6002, Report, China) for 1 h at RT in the dark. Nuclei were stained with DAPI (Cell Signaling Technology, USA). Fluorescence images were captured using a high‐speed confocal microscope (Oxford Instruments, UK) and quantitatively analyzed using ImageJ software (NIH, USA).

For both IHC and IF analyses, liver tissues from *n* = 3 mice per group were used; ≥ 3 non‐adjacent sections per mouse were imaged under identical acquisition/quantification settings, and results were averaged per mouse for statistical analysis with blinding maintained throughout (Chong et al. 2022; Kuramoto et al. 2023).

### 2.11 Western Blot

Liver tissue was lysed on ice with RIPA lysis buffer (Solarbio, China) (containing protease and phosphatase inhibitors), centrifuged to obtain the supernatant, and protein was quantified by BCA (Solarbio, China) method, separated by SDS‐PAGE electrophoresis, and transferred to PVDF membrane (Millipore, USA) by wet transfer method, and blocked with 5% skim milk for 1 h. Western blot analysis was performed on liver samples from *n* = 4 mice per group (biological replicates) (Whitham et al. 2018; Kuramoto et al. 2023). Primary antibodies were incubated overnight at 4°C: IRS1 (1: 2000, AF6273, Affinty, USA), IRS2 (1: 2000, DF7534, Affinty, USA), Akt (1: 2500, HO0915, HuaAnBio, China), p‐Akt (1: 1000, 41,236, arigo, China), Glut4 (1:5000, R1402‐3, HuaAnBio, China), α‐SMA (1: 1000, AF1032, Affinty, USA), Collagen I (1: 500, AF0134, Affinty, USA), Collagen III (1: 1000, AF0136, Affinty, USA), TGF‐β1 (1: 1000, 52,011, arigo, China), Smad2/3 (1: 500, 0034400201, Abclonal, China), p‐Smad (1: 500, AF3367, Affinty, USA), SIRT1 (1: 150, WL02995, Wanleibio, China), LC3I/II (1: 1000, WL01506, Wanleibio, China), P62 (1: 1000, PM045, MBL, Japan), *E*‐cadherin (1: 1000, WL01482, Wanleibio, China), Vimentin (1: 500, WL01960, Wanleibio, China), NAMPT (1: 10000, ARC53731, Abclonal, China), TSG101 (1: 1000, ET1701‐59, HuaAnBio, China), Calnexin (1: 1000, WL03062, Wanleibio, China). After washing with TBST, the membranes were incubated with HRP‐conjugated secondary antibodies either goat anti‐rabbit IgG (1: 5000, S1001, Report, China) or goat anti‐mouse IgG (1: 10000, S1003, Report, China) at room temperature for 1 h. Immunoreactive bands were visualized using ECL chemiluminescence. The grayscale values of the bands were analyzed using Image Lab, and α‐tubulin (1: 5000, 19 U71, ARR3, China) was used as an internal reference for standardization.

### 2.12 Monodansylcadaverine (MDC) Staining

After treatment with normal complete culture medium, the cells were washed with PBS, 1 mL of MDC staining solution (Report, RK1005, China) was added to each well and incubated at 37°C in the dark for 30 min; the staining solution was discarded, and the cells were washed three times with 0.8–1 mL assay buffer, and finally, 1 mL fresh assay buffer was added to cover the cells; the green fluorescence signal was observed and recorded through the ultraviolet excitation channel of the fluorescence microscope to evaluate the formation of autophagic vesicles.

### 2.13 Intracellular NAD ^+^ Measurement and SIRT1 Activity Assay

Based on the manufacturer's instructions (Beyotime, S0175, China), intracellular NAD^+^ and NADH levels were measured using an NAD^+^/NADH assay kit (WST‐8 method). Briefly, cells (1 × 10^6^per sample) were lysed in 200 μL of ice‐cold NAD^+^/NADH extraction buffer by pipetting. The lysates were centrifuged at 12000 g for 10 min at 4°C, and the supernatant was collected for analysis. For total NAD^+^/NADH measurement, 20 μL of the supernatant was transferred to a 96‐well plate. For NADH measurement, the supernatant was heated at 60°C for 30 min to decompose NAD^+^, and then 20 μL was used. Subsequently, 90 μL of alcohol dehydrogenase working solution was added to each well, mixed gently, and incubated at 37°C for 10 min in the dark. Then, 10 μL of chromogenic reagent was added, followed by incubation at 37°C for 30 min in the dark. Absorbance was measured at 450 nm using a microplate reader.

SIRT1 protein levels were measured using a mouse SIRT1 enzyme‐linked immunosorbent assay (ELISA) kit (mlbio, ml037854, China) according to the manufacturer's instructions. Briefly, cells were washed with PBS and lysed by repeated freeze–thaw cycles in PBS to release intracellular components. The lysates were centrifuged at 3000 rpm for 20 min at 4°C, and the supernatant was collected. For the assay, 40 μL of sample dilution buffer and 10 μL of the supernatant were added to 96‐well microtiter plates pre‐coated with anti‐SIRT1 antibody, resulting in a 5‐fold dilution of the sample. The plates were incubated at 37°C for 30 min, washed five times, and then incubated with HRP‐conjugated detection antibody for 30 min at 37°C. After washing, chromogen solutions A and B were added and incubated at 37°C in the dark for 15 min. The reaction was stopped by adding stop solution, and absorbance was measured at 450 nm. SIRT1 concentrations were calculated by comparing the sample OD values to a standard curve.

### 2.14 Statistical Analysis

The experimental data were presented as mean ± SEM and statistically analyzed using GraphPad Prism 9.5. Prior to parametric testing, data were assessed for normality and homogeneity of variance. Two‐way ANOVA combined with Tukey's multiple comparison test was used to analyze differences between multiple groups with two independent variables, and *p* < 0.05 indicated that the difference was statistically significant.

## 3 Results

### 3.1 Exercise Ameliorates HFD‐Induced Liver Metabolic Dysfunction in Aged Mice

To evaluate the therapeutic potential of exercise in the aged MASLD mouse, we subjected mice to a HFD for 4 or 8 months, with or without a concurrent exercise training regimen (Figure 1A). Prolonged HFD feeding induced a time‐dependent exacerbation of hepatic and metabolic dysfunction. Both the 4‐month (HFD 4 M) and 8‐month (HFD 8 M) HFD groups showed a significant increase in body weight (Figure 1B) and the mass of epididymal (eWAT) and inguinal (iWAT) white adipose tissue depots compared to chow‐fed controls (Figure 1C,D). The hypertrophy of iWAT was particularly pronounced in the HFD 8 M group, indicating progressive adiposity. Exercise intervention effectively enhanced physical performance, demonstrated by an increased time to exhaustion and a greater running distance (Figure 1E,F). Concomitantly, exercise attenuated HFD‐induced weight gain and reduced the accumulation of adipose tissue.

Histological analysis revealed that prolonged HFD feeding induced hepatic injury, including hepatocellular ballooning and architectural distortion. This progressive damage was corroborated by a significant increase in lipid accumulation (Oil Red O staining) and a marked elevation in the senescent cell burden (SA‐β‐gal staining) in the HFD 8 M group. Notably, the HFD 8 M mice exhibited nearly a 2.0‐fold increase in SA‐β‐gal‐positive hepatocytes compared to chow‐fed controls (Figure 1G–I). While exercise intervention partially attenuated steatosis and reduced senescence, it failed to fully restore normal hepatic architecture, especially in the HFD 8 M cohort.

Serum levels of ALT and AST were significantly elevated in the HFD 8 M group compared to both the HFD 4 M and Chow control groups (Figure 1J,K). Exercise intervention significantly attenuated this increase in the Ex+HFD 8 M group; however, transaminase levels remained elevated above the baseline of the Chow group, indicating persistent hepatocellular injury despite overall metabolic improvement.

Collectively, these results demonstrate that prolonged HFD exposure in aged mice induces a time‐dependent exacerbation of hepatic steatosis, inflammation, and cellular senescence. Although exercise confers substantial metabolic and functional benefits, particularly in the early stages of the disease, its efficacy in reversing advanced histopathological damage is limited. This underscores the critical importance of early therapeutic intervention for managing aging‐associated MASLD.

### 3.2 Exercise Mitigates Long‐Term HFD Induced Glucose Metabolic Dysfunction and Insulin Signaling Impairment in Aged Mice

**FIGURE 2:**
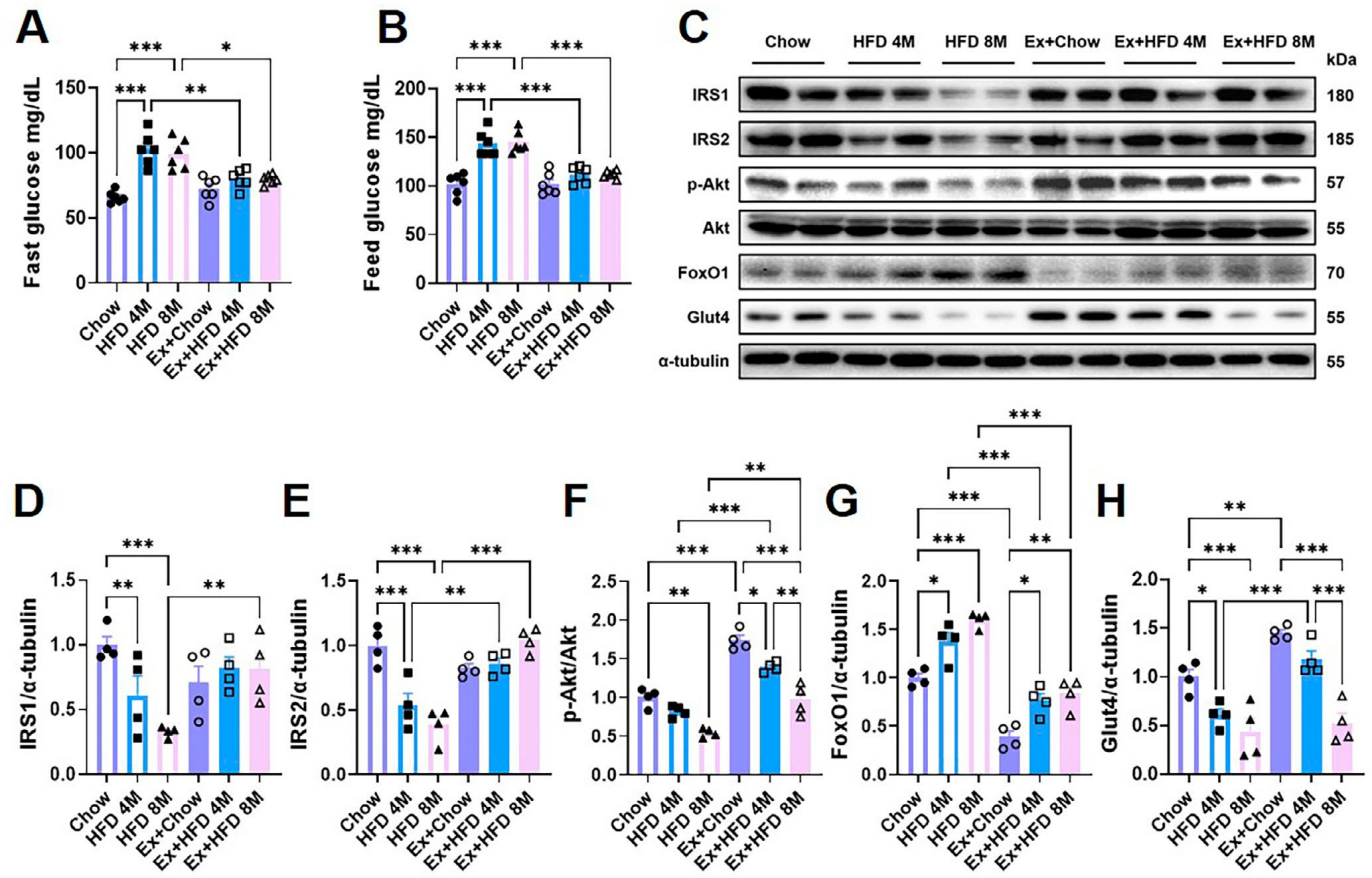
Effects of high‐fat diet and exercise intervention on glucose metabolism and hepatic insulin signaling in aged mice. (A, B) Fasting and postprandial blood glucose levels in aged mice subjected to high‐fat diet (HFD) feeding with or without exercise intervention (*n* = 6). (C‐H) Representative immunoblots and quantification of hepatic insulin signaling components, including IRS1, IRS2, phosphorylated AKT (p‐AKT), total AKT, FoxO1, and Glut4 (*n* = 4). Data are presented as mean ± SEM. Statistical analysis was performed using two‐way ANOVA followed by Tukey's multiple‐comparisons test. *p < 0.05, **p < 0.01, ***p < 0.001.

Chronic HFD feeding induced progressive impairments in glucose metabolism and hepatic insulin signaling in aged mice. Both fasting and postprandial blood glucose levels were significantly elevated in the HFD 4 M and HFD 8 M groups compared to chow‐fed controls (Figure 2A,B). Western blot analysis revealed a time‐dependent disruption of the hepatic insulin signaling pathway (Figure 2C). The expression of key insulin receptor substrates, IRS1 and IRS2, was markedly reduced in HFD fed livers, with the HFD 8 M group exhibiting an approximately 60% depletion compared to controls (Figure 2D,E). Concordantly, the insulin‐stimulated p‐AKT/AKT ratio was diminished by nearly 50% in the HFD 8 M group (Figure 2F). In parallel, the expression of FoxO1, a key transcription factor that promotes gluconeogenesis, was significantly upregulated in both HFD fed groups (Figure 2G). Furthermore, hepatic expression of GLUT4, a critical mediator of insulin‐dependent glucose uptake, was markedly reduced, particularly in the HFD 8 M mice, indicating severely impaired hepatic insulin responsiveness (Figure 2H).

Exercise intervention partially restored insulin signaling. In both the Ex+HFD 4 M and Ex+HFD 8 M groups, fasting and postprandial blood glucose levels were significantly reduced, coinciding with a partial recovery of IRS1/2 expression, the p‐AKT/AKT ratio, and GLUT4 levels (Figure 2A–H). However, this restoration was markedly more robust in the Ex+HFD 4 M group. Insulin signaling pathways remained substantially impaired in the Ex+HFD 8 M mice, indicating a diminished therapeutic responsiveness to exercise in advanced stages of the disease.

Our findings demonstrate that long‐term HFD feeding in aged mice drives a progressive deterioration of glucose homeostasis and insulin signaling. Although exercise effectively mitigates these metabolic abnormalities by enhancing IRS expression and AKT activation, its therapeutic efficacy is significantly attenuated following prolonged metabolic stress. This highlights the critical importance of early lifestyle intervention for the management of age‐related MASLD.

### 3.3 Exercise Restores Hepatic Autophagy Activity via Activating the SIRT1 Signaling Pathway in Aged HFD Fed Mice

**FIGURE 3:**
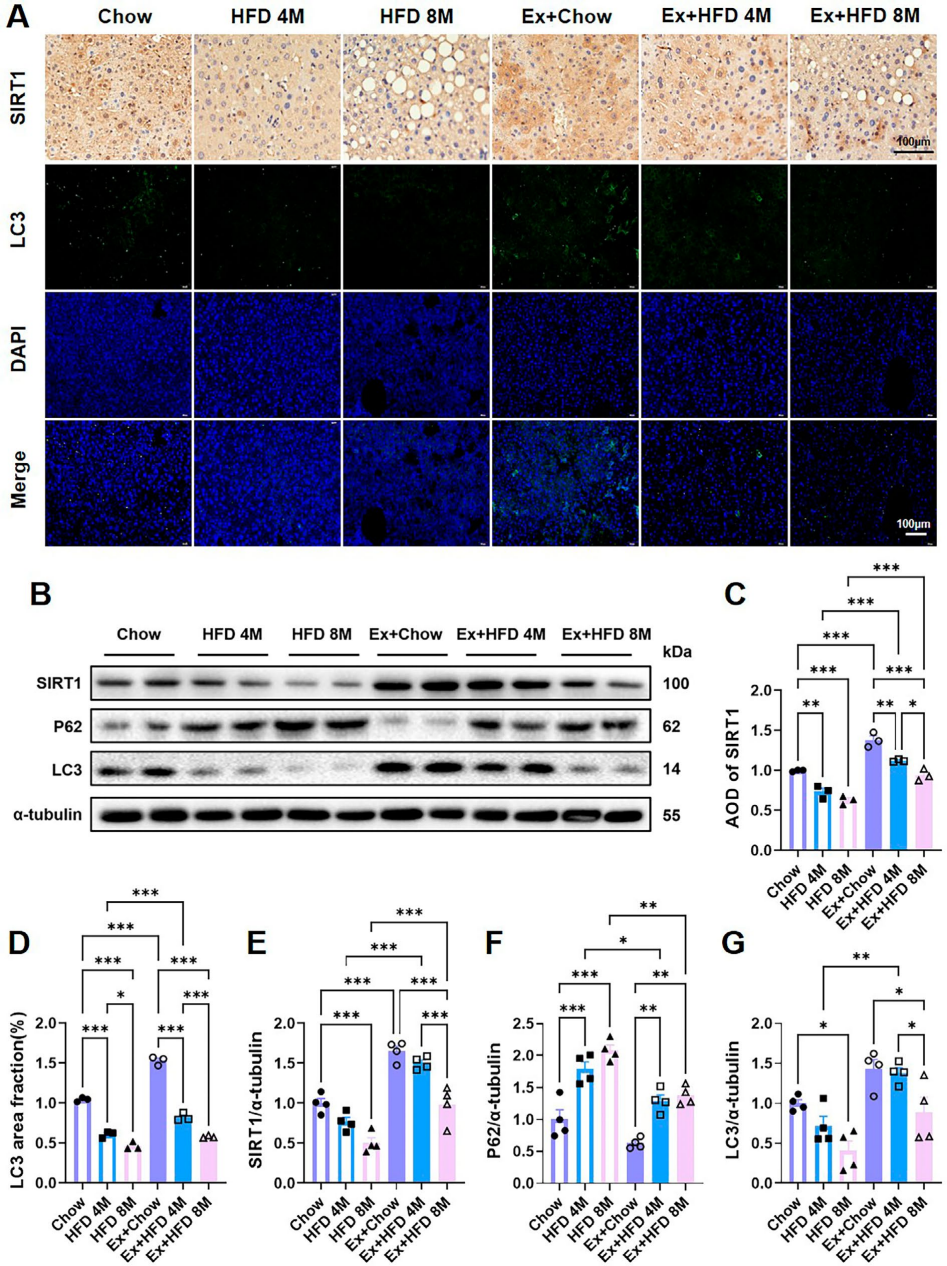
Effects of high‐fat diet and exercise intervention on hepatic autophagy in aged mice. (A) Representative images of SIRT1 immunohistochemistry and LC3 (100× objective) immunofluorescence staining in liver sections from aged mice. (B) Western blot analysis of autophagy‐related proteins in liver tissue. (C, D) Quantification of SIRT1‐positive cells and LC3 fluorescence intensity from immunohistochemistry and immunofluorescence analyses, respectively (*n* = 3). (E–G) Densitometric quantification of hepatic SIRT1, P62, and LC3 protein levels from Western blot data (*n* = 4). Data are presented as mean ± SEM. Statistical significance was determined by two‐way ANOVA followed by Tukey's multiple‐comparisons test. *p < 0.05, **p < 0.01, ***p < 0.001.

Immunohistochemical analysis revealed strong nuclear SIRT1 expression in hepatocytes from chow‐fed mice, which declined progressively with prolonged HFD exposure (Figure 3A,C). Concurrently, LC3 puncta‐indicative of autophagosome formation—were abundant in control livers but were substantially diminished in HFD‐fed mice, with the most pronounced reduction observed in the HFD 8 M group (Figure 3A,D). Western blot analysis corroborated these findings. Protein levels of both SIRT1 and LC3 (LC3‐II) were significantly reduced in HFD 8 M livers compared to chow controls. Conversely, p62, an autophagic substrate that accumulates upon impaired flux, was markedly elevated in both HFD‐fed groups (Figure 3B,E–G). Collectively, these data indicate a time‐dependent HFD‐induced disruption of autophagic turnover.

Exercise intervention significantly upregulated nuclear SIRT1 expression and restored LC3 puncta density in Ex+HFD mice relative to their sedentary counterparts (Figure 3A,C,D). At the protein level, SIRT1 and LC3‐II levels were significantly increased, while p62 accumulation was reduced in exercised mice, suggesting a reactivation of hepatic autophagic flux (Figure 3B,E–G). However, this restoration was incomplete in the Ex+HFD 8 M group, as SIRT1 and LC3 expression remained lower than in the Ex+HFD 4 M group. This indicates that the efficacy of exercise to activate autophagy is attenuated under conditions of prolonged metabolic stress.

These results demonstrate that long‐term HFD feeding impairs hepatic autophagy in aged mice through suppression of the SIRT1 signaling axis. Furthermore, we show that exercise mitigates this impairment by reactivating SIRT1 and restoring autophagic function, albeit with reduced efficacy in advanced disease stages.

### 3.4 Exercise Attenuates HFD Induced Epithelial‐Mesenchymal Transition in Aged Mice

EMT is a key biological process through which epithelial cells acquire mesenchymal characteristics, including migratory and invasive properties. To assess the impact of prolonged HFD feeding and exercise on hepatic EMT progression in aged mice, we analyzed the expression of established EMT markers, Vimentin (mesenchymal) and *E*‐cadherin (epithelial).

**FIGURE 4:**
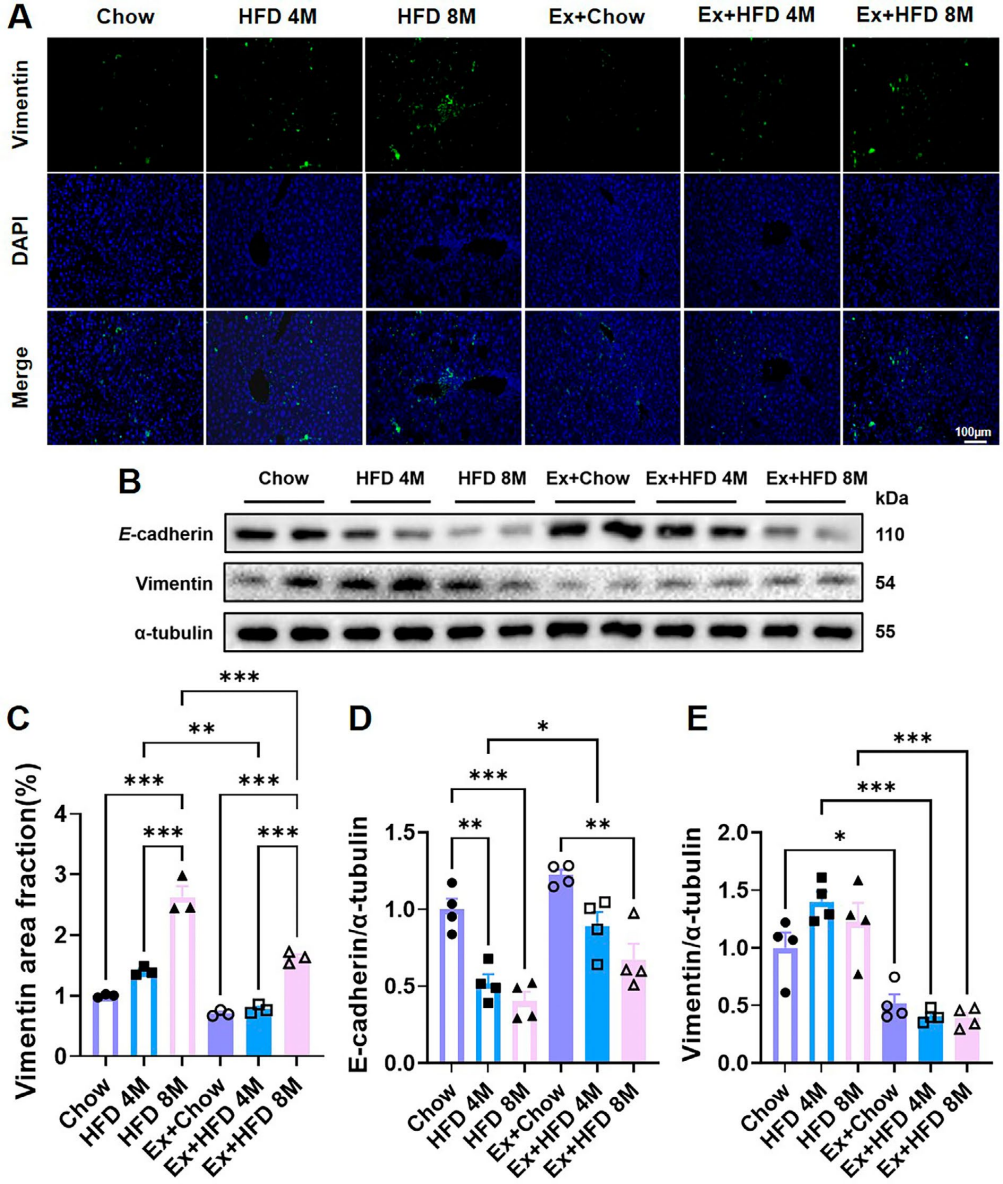
Effects of high‐fat diet and exercise intervention on hepatic epithelial‐mesenchymal transition (EMT) in aged mice. (A) Representative immunofluorescence staining of Vimentin in liver sections from aged mice under high‐fat diet (HFD) feeding with or without exercise intervention. Scale bars: 100 μm (100× objective). (B) Western blot of *E*‐cadherin and Vimentin protein expression in liver tissue. (C) Quantitative analysis of Vimentin immunofluorescence intensity (*n* = 3). (D‐E) Densitometric quantification of hepatic *E*‐cadherin and Vimentin protein levels based on Western blot data (*n* = 4). Data are presented as mean ± SEM. Statistical significance was determined by two‐way ANOVA followed by Tukey's multiple‐comparisons test. *p < 0.05, **p < 0.01, ***p < 0.001.

Immunofluorescence analysis revealed a significant upregulation of Vimentin in the livers of both HFD 4 M and HFD 8 M groups compared to chow‐fed controls, with a more pronounced elevation in the HFD 8 M group, indicating a time‐dependent enhancement of EMT (Figure 4A,C). Exercise intervention effectively suppressed Vimentin expression in the Ex+HFD 4 M group but only partially reduced it in the Ex+HFD 8 M group. This differential effect suggests that prolonged metabolic stress promotes a more stable and potentially irreversible EMT phenotype.

Consistent with the immunofluorescence results, western blot analysis confirmed that exercise intervention significantly increased *E*‐cadherin expression and decreased Vimentin protein levels (Figure 4B,D,E). These restorative effects were more pronounced in the Ex+HFD 4 M group than in the Ex+HFD 8 M group, further supporting the conclusion that exercise more effectively mitigates EMT during the earlier stages of disease progression.

Collectively, these findings indicate that chronic HFD feeding promotes progressive hepatic EMT in aged mice. We demonstrate that exercise can attenuate this process by restoring epithelial marker expression and suppressing mesenchymal marker transition. However, its therapeutic efficacy is significantly diminished following prolonged HFD exposure, highlighting a critical time window for effective intervention.

### 3.5 Exercise Mitigates Liver Fibrosis Induced by Long‐Term HFD in Aged Mice

**FIGURE 5:**
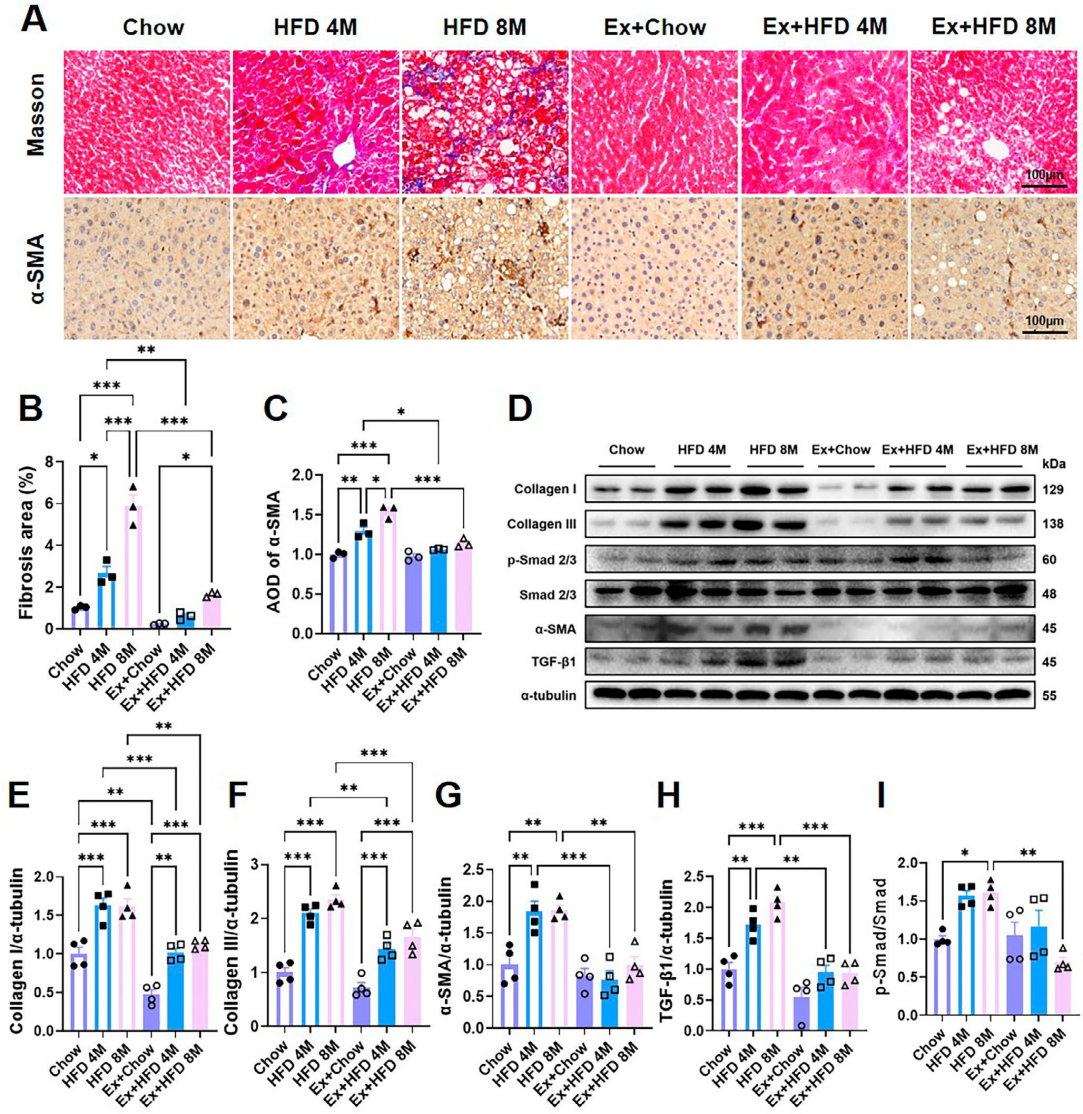
Effects of high‐fat diet and exercise intervention on hepatic fibrosis and TGF‐β1/Smad signaling in aged mice. (A‐C) Representative images and quantification of Masson trichrome staining and α‐smooth muscle Actin (α‐SMA) immunohistochemistry in liver tissue (*n* = 3). (D‐I) Western blot analysis densitometric quantification of fibrosis‐related indicators and TGF‐β1/Smad pathway components, including Collagen I, Collagen III, α‐SMA, TGF‐β1, phosphorylated Smad2/3 (p‐Smad), and total Smad in liver tissue (*n* = 4). Data are presented as mean ± SEM. Statistical significance was determined by two‐way ANOVA followed by Tukey's multiple‐comparisons test. *p < 0.05, **p < 0.01, ***p < 0.001.

To evaluate the impact of prolonged HFD feeding and exercise on hepatic fibrosis, collagen deposition was assessed by Masson's trichrome staining. Livers from chow‐fed control mice exhibited normal histoarchitecture with minimal collagen accumulation. In contrast, HFD 4 M mice displayed mild perivascular collagen deposition and the formation of fibrous septa in portal areas. These fibrotic features were markedly exacerbated in HFD 8 M mice, evidenced by prominent collagen fibers extending into the liver lobules and parenchyma, resulting in significant architectural disruption (Figure 5A,B). Consistent with these findings, immunohistochemical analysis for α‐SMA—a marker of activated hepatic stellate cells (HSCs)—revealed α‐SMA‐positive myofibroblasts confined to portal regions in HFD 4 M mice. In the HFD 8 M group, however, these activated cells were diffusely distributed throughout the liver lobules, indicating widespread HSC activation and progressive fibrosis (Figure 5A,C).

Consistent with histological findings, western blot analysis revealed a significant upregulation of hepatic fibrotic markers—including collagen I, collagen III, and α‐SMA—in both HFD‐fed groups, with the most pronounced elevation observed in the HFD 8 M group (Figure 5D–G). Furthermore, the expression of TGF‐β1 and the phosphorylation of Smad2/3 (p‐Smad2/3), the canonical effectors of pro‐fibrotic signaling, were markedly increased in HFD fed livers (Figure 5D,H,I), confirming activation of the TGF‐β1/Smad pathway.

Exercise intervention significantly attenuated these fibrotic changes. Histological analysis showed that exercise reduced collagen deposition and restricted α‐SMA‐positive cells to periportal areas (Figure 5A–C). At the molecular level, exercise downregulated the expression of collagen I/III, α‐SMA, TGF‐β1, and p‐Smad2/3 (Figure 5D–I), indicating a suppression of HFD‐induced stellate cell activation and fibrogenic signaling.

Collectively, these results demonstrate that long‐term HFD feeding drives progressive hepatic fibrosis in aged mice via activation of the TGF‐β1/Smad signaling axis. Importantly, exercise intervention effectively disrupts this pro‐fibrotic cascade, mitigating collagen accumulation and preserving hepatic architecture, with greater efficacy observed at earlier disease stages.

### 3.6 Exercise‐Derived Exosomal eNAMPT Promotes Autophagy Through SIRT1 Activation in Fatty Liver Cells

**FIGURE 6:**
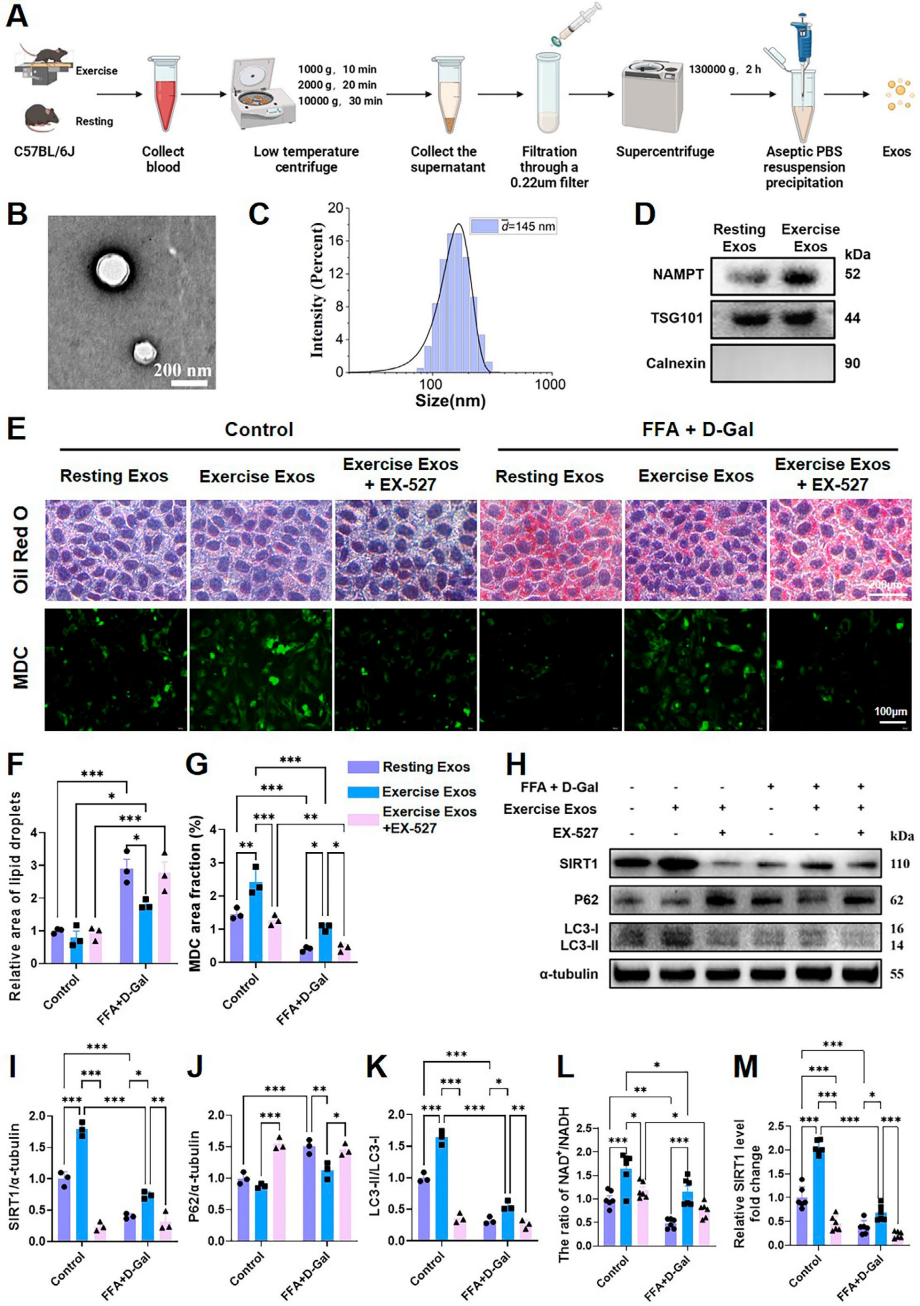
Characterization of exercise‐induced exosomes and activation of the SIRT1‐autophagy axis in vitro. (A) Schematic of the exosome isolation workflow following exercise. (B) Transmission electron microscopy (TEM) image showing the morphology of isolated exosomes. (C) Nanoparticle tracking analysis of exosome size distribution. (D) Western blot analysis confirming exosomal markers TSG101, eNAMPT and Calnexin. (E) Representative images of Oil Red O staining in AML12 cells and MDC staining in primary hepatocytes following 24‐h treatment with free fatty acids (FFA) and D‐galactose (Scale bars: 100 μm, 200× objective). (F, G) Quantitative analysis of intracellular lipid accumulation (Oil red O) and autophagic vesicle formation (MDC) staining in AML12 cells and primary hepatocytes, respectively (*n* = 3). (H‐K) Western blot analysis and densitometric quantification of autophagy‐related proteins in primary hepatocytes treated with exercise‐derived exosomes, with or without the SIRT1 inhibitor EX‐527, including SIRT1, P‐62, LC3‐II/LC3‐I in primary hepatocytes (*n* = 3). (L) The ratio of NAD+/NADH in AML12 cells co‐cultured with exercise‐derived exosomes (*n* = 6). (M) Relative SIRT1 level fold change in AML12 cells co‐cultured with exercise‐derived exosomes (*n* = 6). Data are presented as mean ± SEM. Statistical significance was determined by two‐way ANOVA followed by Tukey's multiple‐comparisons test. *p < 0.05, **p < 0.01, ***p < 0.001.

To elucidate the hepatoprotective mechanism of exercise, we conducted in vitro experiments to investigate exosome‐mediated signaling. Serum exosomes were isolated via ultracentrifugation from C57BL/6J mice subjected to either a single bout of acute exercise or resting conditions (Figure 6A). Characterization confirmed the successful isolation of exosomes: transmission electron microscopy revealed vesicles with a typical cup‐shaped morphology and a double‐layer membrane, with diameters ranging from 30–150 nm (Figure 6B). Nanoparticle tracking analysis indicated a mean particle size of approximately 145 nm, consistent with established exosomal characteristics (Figure 6C). Crucially, western blot analysis demonstrated that eNAMPT was markedly enriched in exosomes derived from exercised mice compared to those from resting controls (Figure 6D).

To model aging‐associated hepatic lipotoxic stress, AML12 cells and primary hepatocytes were treated with D‐galactose (D‐Gal) and free fatty acids (FFA). Oil Red O staining revealed significant intracellular lipid accumulation in the D‐Gal + FFA model group. This accumulation was substantially reduced by treatment with exercise‐derived exosomes (Exercise‐Exos), an effect that was abolished by co‐treatment with the SIRT1 inhibitor EX‐527 (Figure 6E,F).

We further assessed autophagic activity in lipotoxic hepatocytes. In the D‐Gal + FFA model, we observed significant reductions in NAD^+^ content and SIRT1 activity compared with untreated controls, accompanied by suppressed autophagy‐related readouts, including decreased MDC fluorescence, a reduced LC3‐II/LC3‐I ratio, and increased p62 abundance. Treatment with exercise‐Exos restored NAD^+^ and SIRT1 levels and normalized these autophagy‐related markers, as evidenced by increased MDC fluorescence, an elevated LC3‐II/LC3‐I ratio, and decreased p62 accumulation compared with resting exosome controls. Importantly, co‐treatment with EX‐527 completely abrogated the Exercise‐Exos–induced improvements in both NAD^+^/SIRT1 signaling and autophagy‐related markers (Figure 6E,G–M). These findings demonstrate that Exercise‐Exos enhance autophagy‐related responses in lipotoxic hepatocytes through a SIRT1‐dependent mechanism.

Collectively, these in vitro findings support a model in which exercise‐derived exosomes enriched in eNAMPT mitigate lipotoxic stress by activating the NAD^+^–SIRT1 axis, thereby enhancing autophagy in fatty liver cells. This identifies the exosomal eNAMPT‐SIRT1 signaling as a crucial mechanistic pathway contributing to exercise‐mediated hepatoprotection.

### 3.7 Exercise‐Derived Exosomes Inhibit EMT Through SIRT1‐Autophagy Axis

**FIGURE 7:**
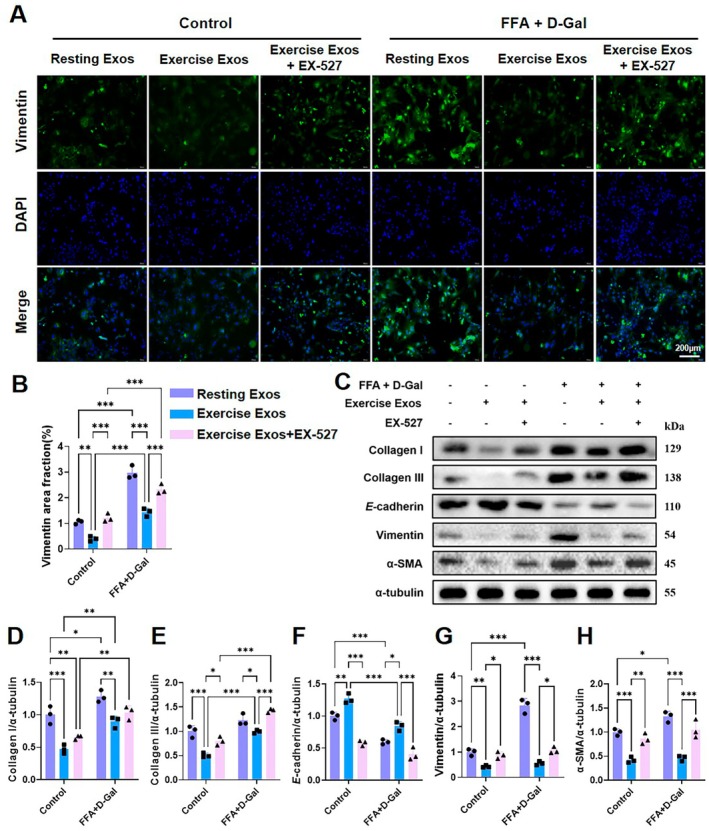
Exercise‐derived exosomes activate autophagy and inhibit epithelial‐mesenchymal transition (EMT) through a SIRT1‐dependent mechanism. (A, B) Immunofluorescence staining and quantitative analysis of Vimentin expression in primary hepatocytes treated with free fatty acids (FFA) and D‐galactose followed by co‐culture with exercise‐derived exosomes (*n* = 3, scale bars: 200 μm, 100× Objective). (C‐H) Western blot analysis and densitometric quantification of EMT and fibrosis‐related proteins, including Collagen I, Collagen III, *E*‐cadherin, Vimentin, and α‐smooth muscle Actin (α‐SMA), in primary hepatocytes under the indicated treatments (*n* = 3). Data are presented as mean ± SEM. Statistical significance was determined by two‐way ANOVA followed by Tukey's multiple‐comparisons test. *p < 0.05, **p < 0.01, ***p < 0.001.

Given the observed restoration of SIRT1 expression and autophagic flux by Exercise‐Exos, we next investigated their impact on EMT in hepatocytes under lipotoxic stress. Compared to control cells, hepatocytes treated with D‐galactose and FFA exhibited a significant increase in Vimentin fluorescence intensity (Figure 7A,B), elevated protein levels of Vimentin and α‐SMA, and reduced expression of *E*‐cadherin (Figure 7C–H), indicative of EMT activation. Treatment with Exercise‐Exos significantly reversed these changes, markedly reducing Vimentin intensity and protein levels, downregulating α‐SMA, and restoring *E*‐cadherin expression relative to Resting‐Exos‐treated cells (Figure 7A–H). Importantly, the beneficial effects of Exercise‐Exos on these EMT markers were abolished by co‐treatment with the SIRT1 inhibitor EX‐527, confirming the SIRT1‐dependence of this mechanism.

Together, these in vitro findings demonstrate that exercise‐derived exosomes inhibit EMT progression in hepatocytes via a SIRT1‐dependent pathway. This provides direct mechanistic support for the role of the exosomal eNAMPT–SIRT1–autophagy axis in mediating the anti‐fibrotic benefits of exercise in MASLD.

## 4 Discussion

**FIGURE 8:**
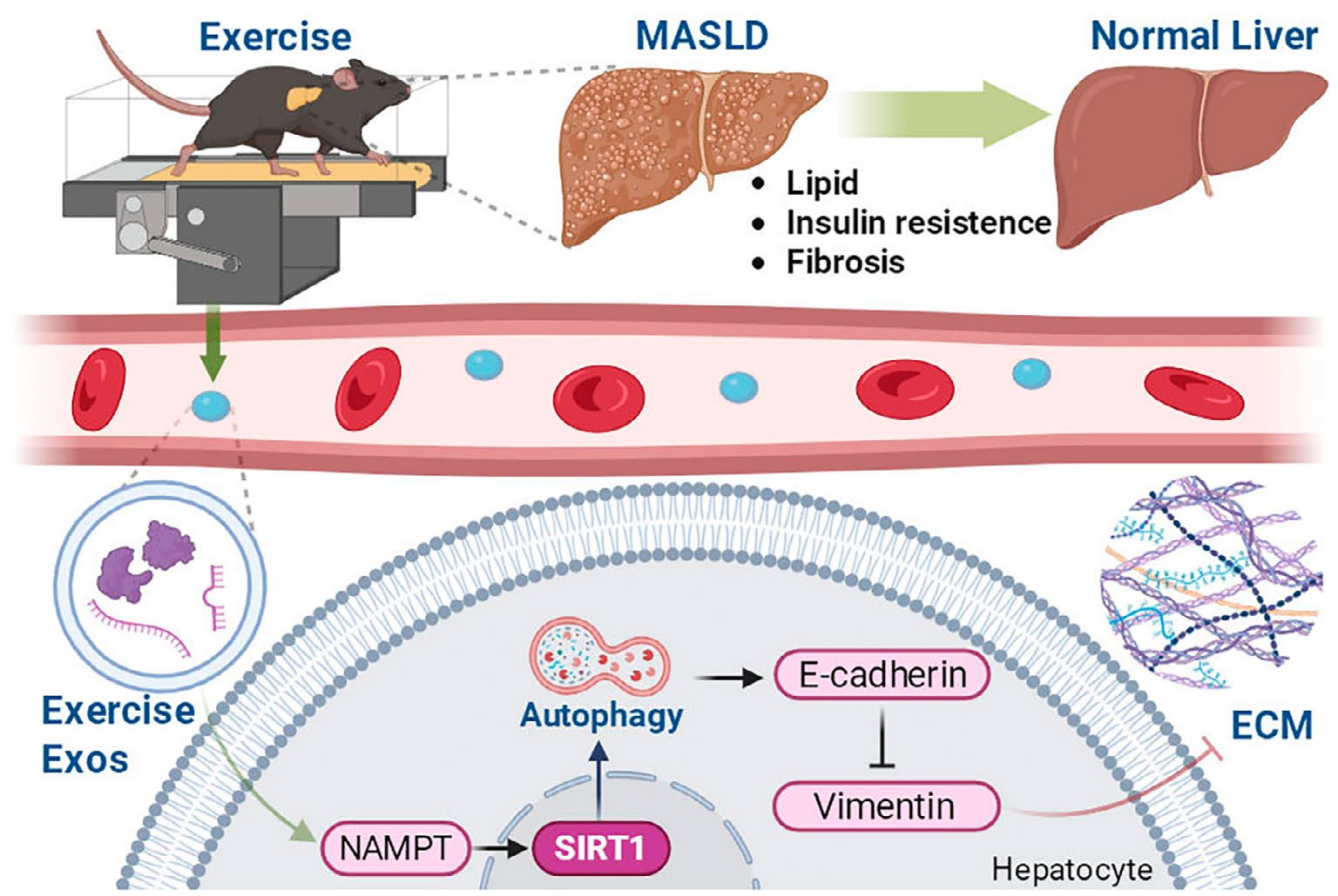
Exercise alleviates age‐related progression of metabolic dysfunction‐associated steatotic liver disease (MASLD) via exosome‐mediated activation of the SIRT1‐autophagy axis.

In this study, we investigated the protective effects and underlying mechanisms of exercise in aged mice with MASLD induced by long‐term HFD feeding. Our findings demonstrate that (i) prolonged HFD exposure progressively exacerbates hepatic steatosis, insulin resistance, fibrotic remodeling, and autophagy impairment; (ii) exercise intervention attenuates these pathological features, including suppression of EMT‐associated responses; and (iii) these benefits are conveyed, at least in part, by exercise‐induced exosomes enriched in eNAMPT, which activate the NAD^+^‐SIRT1‐autophagy axis. Collectively, our work identifies a novel exosome‐mediated, SIRT1‐dependent mechanism linking exercise to attenuation of age‐related MASLD progression (Figure 8).

To model age‐related MASLD, aged mice were subjected to a HFD treatment for up to 8 months. This regimen induced progressive hepatic lipid accumulation, inflammation, cellular senescence, insulin resistance, and fibrosis. These pathological changes were most severe in the HFD 8 M group, thereby validating a robust, time‐dependent model that recapitulates key features of human MASLD progression.

Consistent with the amelioration of systemic metabolic parameters, exercise intervention effectively alleviated hepatic insulin resistance. This partial recovery of insulin sensitivity was evidenced by the restored expression of IRS1 and IRS2, an increased p‐AKT/AKT ratio, enhanced GLUT4 membrane localization, and suppressed FoxO1 activity. These findings align with the established paradigm wherein insulin resistance promotes de novo lipogenesis and hepatic lipid accumulation (Utzschneider and Kahn 2006). Given that intact insulin signaling is a central regulator of both glucose and lipid homeostasis (Boucher et al. 2014; Saltiel and Kahn 2001), its restoration likely constitutes a primary mechanism through which exercise confers metabolic benefits in the aged, HFD‐challenged liver.

Beyond ameliorating metabolic functions, exercise intervention showed potent anti‐fibrotic effects. We observed a marked reduction in the hepatic expression of the key fibrotic markers α‐SMA and Collagen I/III, concomitant with suppressed activation of the pro‐fibrotic TGF‐β/Smad2/3 signaling pathway. These observations underscore the capacity of exercise to confer dual benefits by alleviating both metabolic dysregulation and structural pathology in the aged MASLD liver.

At the molecular level, our investigations revealed that long‐term HFD feeding significantly impaired hepatic autophagic flux and downregulated SIRT1, a NAD^+^‐dependent deacetylase central to the maintenance of cellular metabolic homeostasis. Autophagic dysfunction was confirmed by a decreased LC3‐II/LC3‐I ratio and the pronounced accumulation of the autophagy substrate p62. Crucially, exercise intervention effectively restored both autophagic activity and SIRT1 expression. Importantly, our in vitro data further extend these in vivo observations by demonstrating that Exercise‐Exos increase intracellular NAD^+^ availability and enhance SIRT1 enzymatic activity, thereby providing functional evidence that exercise‐derived exosomal signaling engages the NAD^+^‐SIRT1 node upstream of autophagy regulation. This coordinated recovery suggests that the reactivation of the SIRT1‐autophagy axis is a principal mechanism underpinning the observed hepatoprotection. Our findings are strongly supported by a body of prior evidence establishing SIRT1 as a master regulator of lipid metabolism, insulin sensitivity, and cellular aging through its modulation of autophagic processes (J. Y. Kim et al. 2022; Kitada and Koya 2013; Schug and Li 2011; Wu et al. 2022).

Further mechanistic dissection identified eNAMPT‐enriched Exercise‐Exos as the principal mediators of this protective axis. As the rate‐limiting enzyme in NAD^+^ biosynthesis, exosomal eNAMPT was sufficient to elevate intracellular NAD^+^ levels, thereby enhancing SIRT1 deacetylase activity. This activation triggered a robust increase in the LC3‐II/LC3‐I ratio and a concomitant reduction in p62 accumulation, confirming the restoration of autophagic flux. Critically, the pharmacological inhibition of SIRT1 with EX‐527 completely abrogated the exosome‐induced activation of autophagy, establishing that the eNAMPT‐NAD^+^‐SIRT1 signaling cascade is indispensable for the observed benefits. Although eNAMPT has been implicated in promoting pathological fibrosis in certain microenvironments (Chen et al. 2024), our findings underscore the profound importance of its context and delivery mechanism. Specifically, when encapsulated within exosomes, eNAMPT elicits protective, homeostatic effects, a conclusion consistent with established literature on NAD^+^‐SIRT1‐autophagy interactions (Chong et al. 2022; He et al. 2021; Peng et al. 2024; Roulston and Shore 2016).

Importantly, the activation of the SIRT1‐autophagy axis by Exercise‐Exos also suppressed the EMT, a fundamental driver of fibrogenesis in MASLD. EMT is characterized by the loss of epithelial markers (e.g., *E*‐cadherin) and the acquisition of mesenchymal markers (e.g., Vimentin), which collectively promote cellular migration and fibrotic transformation (Ribatti et al. 2020). In both our in vivo and in vitro models, treatment with Exercise‐Exos effectively reversed this signature, increasing *E*‐cadherin and decreasing Vimentin expression. Importantly, the SIRT1 inhibitor EX‐527 completely abolished these protective effects, demonstrating a direct causal role for SIRT1. Collectively, our findings support a model wherein Exercise‐Exos deliver eNAMPT to activate the SIRT1‐autophagy axis, which in turn functions to preserve hepatic epithelial identity and suppress EMT‐driven fibrotic progression.

Our findings reinforce the emerging paradigm that exercise‐induced exosomes function as critical systemic mediators of inter‐organ communication, transmitting metabolically beneficial signals independent of direct mechanical stimulation. Exosomes have increasingly been recognized as essential vectors for endocrine‐like signaling, capable of transporting a diverse cargo of proteins, enzymes, RNAs, and metabolites to orchestrate responses in distal tissues (Safdar and Tarnopolsky 2018; Whitham et al. 2018). Our study now positions exosomes as natural nanocarriers for eNAMPT, delivering this key enzyme to the aged liver to mitigate core pathological processes. This mechanism is of paramount relevance in the context of aging, where the confluence of autophagy deficiency, sustained EMT activation, and cellular senescence creates a permissive environment for the accelerated progression of MASLD.

From a translational perspective, our work provides a compelling rationale for developing exosome‐based therapeutic strategies for MASLD, particularly in elderly or sedentary individuals who are unable to engage in sustained exercise regimens. Engineering natural exosomes or harnessing those enriched with specific cargoes like eNAMPT may represent a promising “exercise mimetic” strategy for targeted metabolic reprogramming in the liver. Future studies are warranted to optimize the delivery specificity, stability, and long‐term safety of exosome‐based interventions in vivo.

Nevertheless, several limitations of this study must be acknowledged. First, the precise tissue origin and the complete molecular cargo of the exercise‐induced exosomes used here remain incompletely defined. While eNAMPT was identified as a key effector, it is likely that other exosomal components (e.g., miRNAs, other proteins) act synergistically to contribute to the observed benefits. Second, our findings would be strengthened by direct in vivo administration studies to conclusively confirm the therapeutic efficacy of isolated Exercise‐Exos in aged models of MASLD. Therefore, further research is essential to fully evaluate the clinical applicability of exosome‐based therapies for age‐related metabolic diseases and to delineate the full spectrum of molecular mediators involved.

## Author Contributions

**Wenxuan Song:** data curation, methodology, software, writing – original draft. **Naijun Wu:** data curation, methodology. **Xing Li:** conceptualization, methodology, writing – original draft. **Dan Li:** data curation, methodology. **Jiaqi Li:** data curation, methodology. **Siqi Liu:** methodology. **Zhiwei Yue:** data curation. **Qiang Ma:** data curation. **Xuefeng Zhu:** supervision, resources, and editing. **Yajuan Qi:** supervision, writing – review and editing, funding acquisition.

## Funding

This work was supported by Key research and development Project of Tangshan Science and Technology Plan, 25150212E. Key project of Traditional Chinese medicine Joint Fund of Hebei Province, H2022209087. Hebei yanzhao golden platform top talent program, HY2025100003. Hebei Provincial Medical Science Research Project, 20260636. Hebei Natural Science Foundation, H2021209013, H2018209341. National Natural Science Foundation of China, 81471022. Medical‐Engineering Integration Program of North China University of Science and Technology, ZD‐YG‐202406.

## Conflicts of Interest

The authors declare no conflicts of interest.

## Data Availability

The data that support the findings of this study are available on request from the corresponding author. The data are not publicly available due to privacy or ethical restrictions.
